# The Antitumor Didox Acts as an Iron Chelator in Hepatocellular Carcinoma Cells

**DOI:** 10.3390/ph12030129

**Published:** 2019-09-02

**Authors:** Michela Asperti, Luca Cantamessa, Simone Ghidinelli, Magdalena Gryzik, Andrea Denardo, Arianna Giacomini, Giovanna Longhi, Alessandro Fanzani, Paolo Arosio, Maura Poli

**Affiliations:** Department of Molecular and Translational Medicine, University of Brescia, Viale Europa 11, 25123 Brescia, Italy

**Keywords:** didox, iron chelators, antitumor compound, iron metabolism, RRM2

## Abstract

Ribonucleotide reductase (RR) is the rate-limiting enzyme that controls the deoxynucleotide triphosphate synthesis and it is an important target of cancer treatment, since it is expressed in tumor cells in proportion to their proliferation rate, their invasiveness and poor prognosis. Didox, a derivative of hydroxyurea (HU), is one of the most potent pharmaceutical inhibitors of this enzyme, with low in vivo side effects. It inhibits the activity of the subunit RRM2 and deoxyribonucleotides (dNTPs) synthesis, and it seems to show iron-chelating activity. In the present work, we mainly investigated the iron-chelating properties of didox using the HA22T/VGH cell line, as a model of hepatocellular carcinoma (HCC). We confirmed that didox induced cell death and that this effect was suppressed by iron supplementation. Interestingly, cell treatments with didox caused changes of cellular iron content, TfR1 and ferritin levels comparable to those caused by the iron chelators, deferoxamine (DFO) and deferiprone (DFP). Chemical studies showed that didox has an affinity binding to Fe^3+^ comparable to that of DFO and DFP, although with slower kinetic. Structural modeling indicated that didox is a bidentated iron chelator with two theoretical possible positions for the binding and among them that with the two hydroxyls of the catechol group acting as ligands is the more likely one. The iron chelating property of didox may contribute to its antitumor activity not only blocking the formation of the tyrosil radical on Tyr122 (such as HU) on RRM2 (essential for its activity) but also sequestering the iron needed by this enzyme and to the cell proliferation.

## 1. Introduction

**Figure 1:**
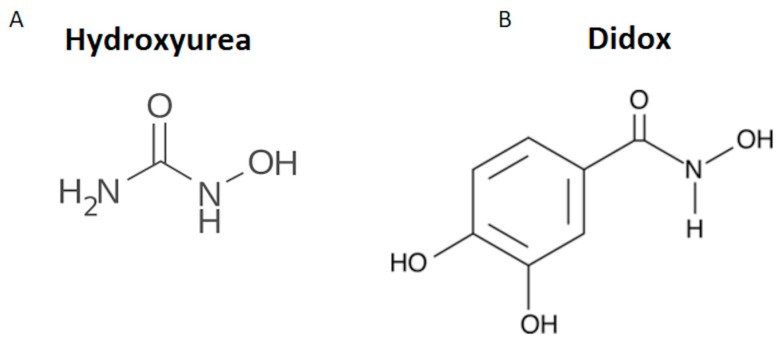
Chemical structure of hydroxyurea and its derivative didox. The structure of hydroxyurea (HU) (**A**) has been modified to obtain that one of didox (**B**) in which the amino group of HU has been substituted by a catechol group.

Ribonucleotide reductase (RR) is one of the fifty genes reported to be overexpressed in highly malignant tumors with poor prognosis [1,2,3,4,5]. RR is essential for DNA synthesis during cell division encoding the rate-limiting enzyme that catalyzes the conversion of ribonucleotides (NTPs) into deoxyribonucleotides (dNTPs) [3,6,7,8]. The enzyme is composed of two catalytic (RRM1) and two regulatory (RRM2 or p53R2) subunits [9]. RRM1 expression is detectable throughout the cell cycle in all tissues [10], while RRM2 and p53R2 are preferentially expressed during cell mitosis and in response to DNA damage, respectively. The reductase activity of RRM2 subunit requires two Fe^3+^ ions for the formation of the tyrosil radical on Tyr122 that has a key role in the enzyme activity [6]. Hydroxyurea (HU), gemcitabine, fludarabine and chlorodeoxydenoside are compounds targeting RRM2 activity that showed effects in cancer therapy in vitro, but with some side effects in vivo. In order to improve their efficacy in the inhibition of RRM2 and reduce the side effects, some derivatives of polyhydroxy-substituted benzohydroxamic have been synthesized [3,11,12] and among them there is 3,4 dihydroxybenzohydroxamic acid (didox) in which the amino group of HU is substituted by a catechol group (Figure 1A,B).

This compound, targeting RRM2 subunits, was found to trigger cell apoptosis with a different extent depending on the cell types [13,14,15] by increasing the levels of the pro-apoptotic protein Bax and release of cytochrome C from the mitochondria [16]. Didox revealed good efficacy against multiple myeloma cells [15], prostate tumor [16], breast cancer cells [17] and acute and chronic myeloid leukemia HL-60 and K562 cell lines [14]. Mouse models have shown that didox significantly caused growth inhibition of breast cancer [17] and human leukemia [18]. Furthermore, in phase I/II clinical trials, didox showed minimal toxicity in cancer patients [19,20].

Interestingly, it was also reported that didox forms iron complexes recognized by photometric methods [21]. The finding that the antiproliferative effect of didox was partially inhibited by iron suggested that iron chelation may be important for its pharmacological activity [21]. Tumor cells are often characterized by an “iron addiction” status requiring abundant iron to sustain proliferation [22,23], thus it has been indicated that iron chelators may improve the chemotherapeutic effects [24,25,26,27,28]. The potential clinical impact of the new generation of iron chelators has increased recently due to the improvement of their pharmacokinetic and pharmacodynamic properties [29]. Iron chelators act to inhibit cell proliferation, by subtracting the iron needed for cellular metabolism, by inducing apoptosis and also by contributing to the generation of reactive oxygen species. This depends largely by the coordination of the chelant, deferoxamine (DFO; Desferal ^®^) is a hexadentate iron chelator that blocks the interaction of iron with oxygen, making it inert. Instead, bidentate chelators such as deferiprone (DFP; Ferriprox ^®^), or the tridentate deferasirox bind iron in a 3:1 or 2:1 chelator to iron ratio, resulting in a less stable iron complex, thus allowing the formation of potentially toxic free radicals [30,31].

The iron binding properties of didox have been described only by Fritzer-Szekeres [21] but its pharmacological effects and its impact on iron metabolism have not been further studied. In the present work we studied the effect of didox on the proliferation of the hepatocellular carcinoma HA22T/VGH cell line and we characterized the iron-chelating properties of didox in vitro and in cells, specifically focusing our study to determine the iron binding capacity of didox and its effect on iron related proteins.

## 2. Results

### 2.1. Didox Suppresses the Viability of Hepatocellular Carcinoma HA22T/VGH Cell Line

**Figure 2:**
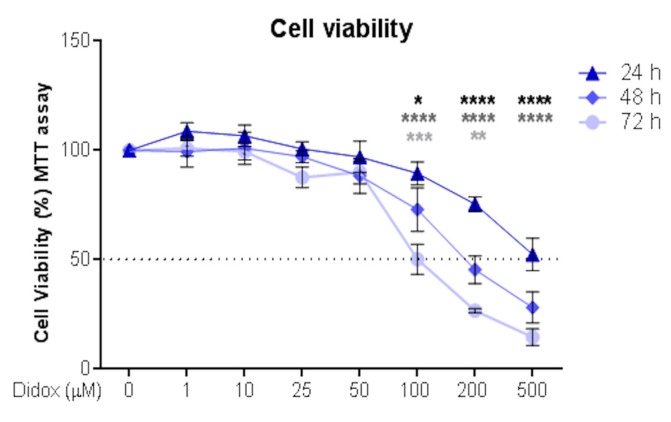
Didox reduced cell viability in HA22T/VGH cell line in time and a dose-dependent manner. HA22T/VGH were treated with 0, 1, 10, 25, 50, 100, 200 and 500 µM of didox for 24 (triangle, blue line), 48 (diamond, blue-sky line) and 72 (circle, light blue line) hours. An MTT assay was performed to verify cell viability after treatment. The values are expressed as % of viable cells over the not treated cells (0) at the indicated time point. The black dot line is drawn in correspondence to the half maximal inhibitory dose (IC_50_). The graph represents the means of three independent experiments (*N* = 3) with three internal values for each experiment. The black stars correspond to the comparison between 24 and 48 h; the grey stars between 24 and 72 h and the light grey stars between 48 and 72 h. * *p* < 0.05; ** *p* < 0.01; *** *p* < 0.001; **** *p* < 0.0001.

The hepatocellular carcinoma (HCC) HA22T/VGH cell line was chosen as a model to study didox antitumor activity. It has a detectable level of RRM2 and high levels of intracellular iron and iron-related proteins, in line with its hepatic origin (not shown). The cells were incubated with different concentrations of didox (1, 10, 25, 50, 100, 200 and 500 µM) for 24–48 and 72 h and then their viability analyzed by an MTT assay. Didox reduced cell viability in dose- and time-dependent manner (Figure 2) with increasing potency at the concentration of 100, 200 and 500 µM and after 48 and 72 h (Figure 2). The half maximal inhibitory dose (IC_50_) at 48 h was 283.36 ± 18.82 µM and at 72 h was 132.98 ± 7.97 µM, indicating that time of exposure is important in this cell line (Table S1).

We confirmed the results with a second HCC cell line, HuH7, with the same doses and time of exposure used for HA22T/VGH and we observed that the sensitivity to the drug was similar in the two HCC cells (Figure 2 and Figure S1) with an IC_50_ for HuH7 similar to that of HA22T/VGH (329.31 ± 31.55 µM at 48 h and 122.92 ± 13.21 µM at 72 h), confirming that time exposure is important in both cell lines (Table S1).

### 2.2. Didox Induces Apoptosis and Increases Mitochondrial ROS

**Figure 3:**
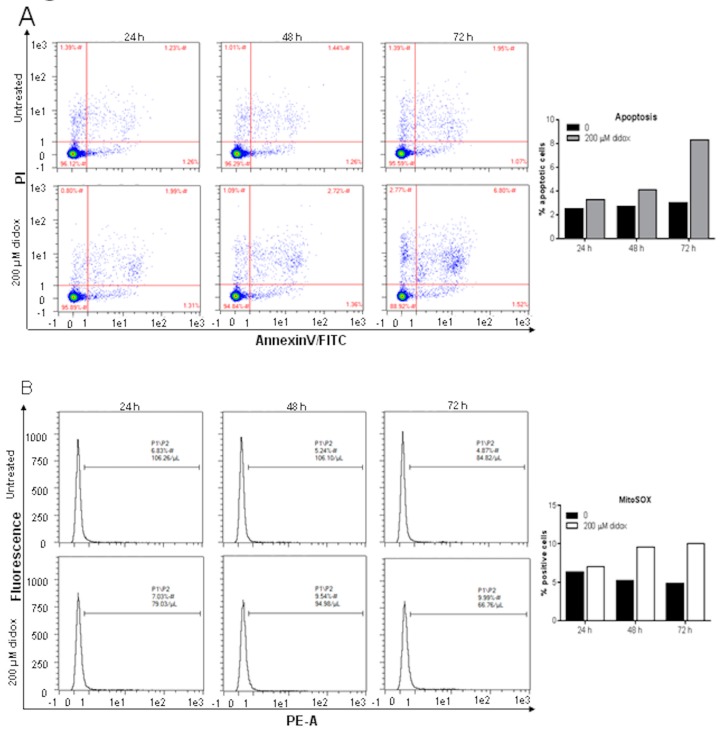
Didox induced apoptotic cell death and mitochondrial oxidative stress in HA22T/VGH cell lines. Cells were untreated or treated with 200 µM of didox for 24, 48 and 72 h. At each time point, cells were analyzed for apoptotic cell death combining AnnexinV/FITC/PI (**A**) or using MitoSOX Red mitochondrial superoxide indicator (**B**) and analyzed by flow-cytometry. The histograms show the percentage of apoptotic cell death, positive to AnnexinV (**A**) or fluorescent cells positive to MitoSOX mitochondrial superoxide indicator (PE-A,) (**B**).

Didox was previously shown to cause cell death by an apoptotic mechanism with an increase of AnnexinV positive cells of about 30–50% after 24–48 h at 250 µM and only at high concentration to cause a little induction of caspase8 and 9 in HL-60 and K562 cells [14,16]. To verify this, we treated HA22T/VGH with 200 µM didox for 24, 48 and 72 h. Then the cells were labeled for AnnexinV-FITC and with propidium iodide (PI) and analyzed with flow-cytometry. Staining cells simultaneously with AnnexinV-FITC and PI allows the discrimination of intact cells (AnnexinV-FITC negative and PI negative), early apoptotic (AnnexinV-FITC positive and PI negative) and late apoptotic or necrotic cells (AnnexinV-FITC positive and PI positive). Didox caused a time dependent increase of apoptotic cells (considering early and late apoptosis) to about 8% after 72 h (Figure 3A).

To detect the level of mitochondrial ROS the HA22T/VGH cells were treated with 200 µM didox for 24, 48 and 72 h and then labeled with a MitoSOX probe and the fluorescence measured on flow-cytometry. This probe is used for the selective detection of superoxide in the mitochondria in fact, once in the mitochondria; it is oxidized by superoxide and shows red fluorescence. Didox caused an increase of MitoSOX fluorescence of about 10–12% after 48–72 h meaning an increase of mitochondrial ROS levels (Figure 3B). In parallel experiments, we found that the iron (III) chelator DFO induced similar increases of AnnexinV positive cells and mitochondrial ROS in this cell line (not shown).

### 2.3. Didox Inhibits Cell Viability Similarly to DFO and DFP

**Figure 4:**
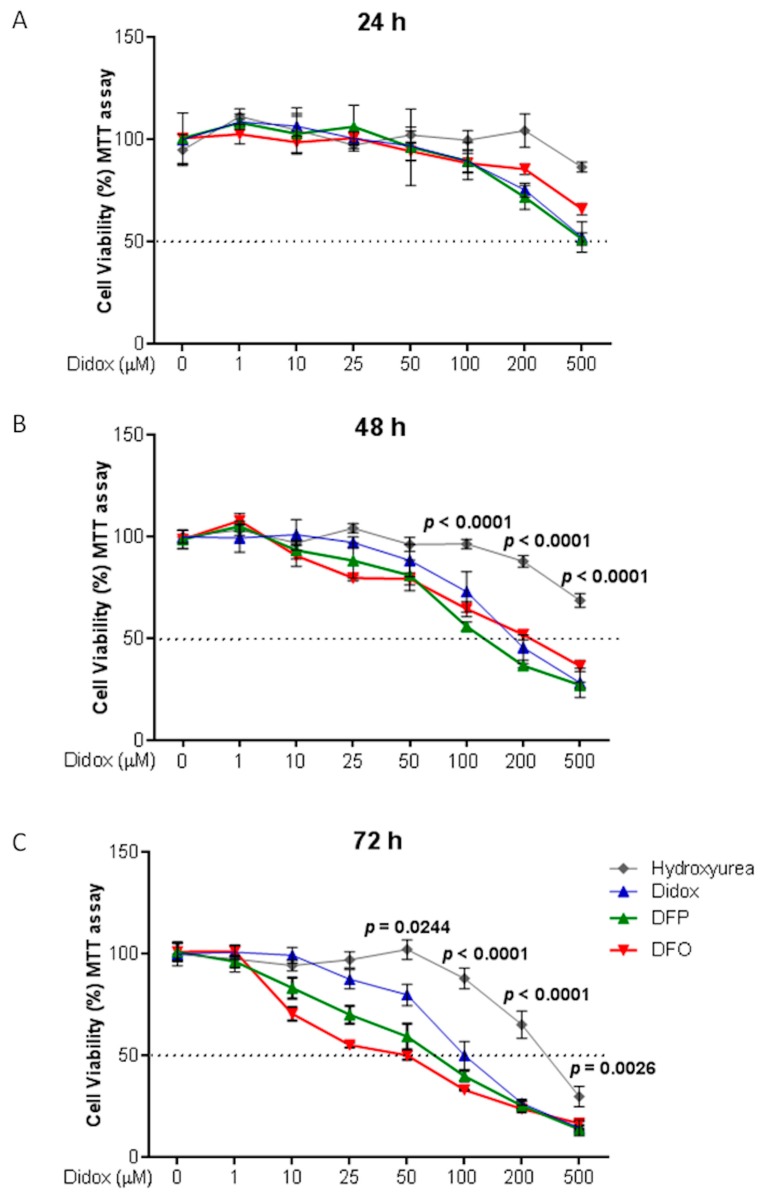
Didox was more effective than hydroxyurea in reducing cell viability in HA22T/VGH. HA22T/VGH cells were treated with 0, 1, 10, 25, 50, 100, 200 and 500 µM of didox (blue lines) or HU (grey lines) or deferoxamine (DFO; red lines) or deferiprone (DFP; green lines) for 24 (**A**), 48 (**B**) and 72 h (**C**). MTT assay was performed to verify cell viability after treatment. The values are expressed as % of viable cells over the not treated cells (0) at the indicated time point. The graphs represent the means of three independent experiments (*N* = 3) with three internal values for each experiment and statistic obtained comparing with each other the efficacy of the compounds at a certain dose of treatment. The black dot line is drawn in correspondence to the half maximal inhibitory dose (IC_50_).

It is known that the iron chelators cause cell death sequestering the iron essential for the cell proliferation. To compare the potency to inhibit cell viability of didox to its precursor (HU) and other well known chelators (such as DFO and DFP), HA22T/VGH cells were treated with increasing concentrations of didox, HU, DFO and DFP (1, 10, 25, 50, 100, 200 and 500 µM) for 24–48 and 72 h, and then cell viability was monitored using MTT assay. HU was the least potent of the four compounds with an IC_50_ of about 400 µM at 72 h, that was about four-fold higher than that of didox, DFO and DFP (of about 100 µM at 72 h; Figure 4A–C). Didox, DFO and DFP showed a very similar time-dependent activity that is possibly due to the progressive chelation of intracellular iron and the inhibition of different cellular activity.

### 2.4. Didox Binds Fe^3+^ in a Time-Dependent Manner

As shown in the Figure 4, didox had higher efficacy than HU in inhibiting HA22T/VGH cell viability, a property that may be due to the didox iron-binding capacity, which seems to be absent in HU (Figure 4A–C). To elucidate this point, we used the in vitro calcein assay. Calcein is a fluorescent probe, which its signal is quenched by the binding to iron (II) as is well reported in the paper of Breuer W. [32]. Only the addition of iron (III) chelators (such as DFO and DFP) can restore the fluorescence of the probe, removing iron from the iron-calcein complex [32].

**Figure 5:**
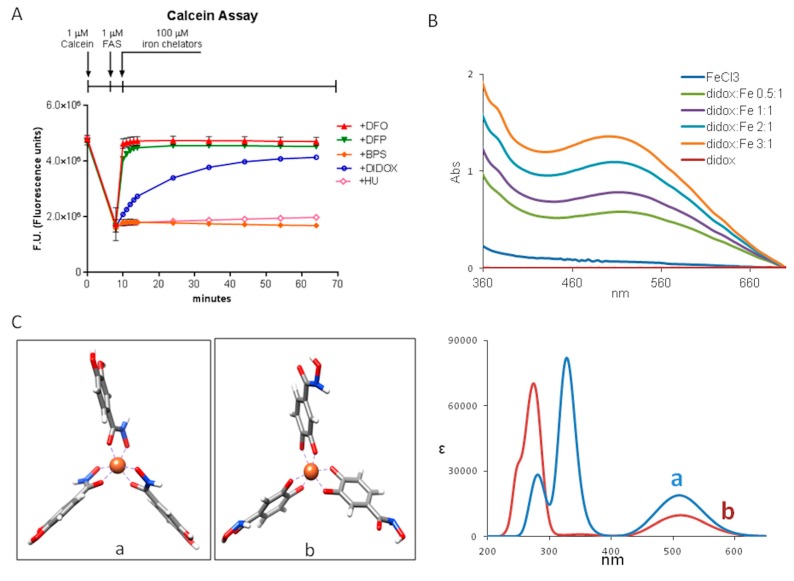
Didox retains the capacity to dequench the fluorescence of calcein forming a complex with iron (III). (**A**) The fluorescence of 1 µM calcein was determined, to set the basal level of the fluorescence at the beginning of the assay. Iron (II), as 1 µM FAS (ferrous ammonium sulfate), was added and allowed to equilibrate and form calcein-iron complexes for 8 min. Subsequently, 100 µM iron (III) chelators (such as DFO, red lines; DFP, green line), 100 µM iron (II) chelator (BPS, orange line), 100 µM didox (blue line) or 100 µM HU (pink line) were added. The fluorescence was measured after 1, 2, 3, 4, 5, 15, 25, 35, 45, 55 and 65 min after the addition of the compounds. (**B**) UV-vis spectra for complexes formed by base titration at constant concentration FeCl3 (300 µM) with increasing concentrations (0–900 µM) of didox in 25 mM Tris-HCl pH 7.2 buffer. (**C**) Left: Didox complexed with iron in the hydroxamic type of chelate (a) and catechol type of chelate (b). Right: Calculated UV-vis spectra of didox-iron complex a and b.

Iron (II) as ferrous ammonium sulfate (1 µM) was added to calcein (1 µM) at a 1:1 molar ratio causing 60% fluorescence quenching. Then the chelators were added. The well-characterized iron (III) chelators DFO and DFP (100 µM) caused a fast and complete dequenching, while HU (100 µM) and also bathophenantroline disulfonic acid (BPS, 100 µM) had no evident effect and didox (100 µM) had an intermediate behavior, slowly causing fluorescence dequenching that was almost complete after one hour (Figure 5A).

Then we incubated the colorless didox with increasing amounts of iron (III) salts. This produced a colored component with a maximum absorbance at 510 nm attributed to the didox-iron complex (Figure 5B). Next we carried out density functional theory (DFT) calculations to test the possible structures, as done before in the study of similar ferric complex structures [33]. The results indicate that didox acts as a bidentate ligand that can chelate iron either through the hydroxaminic group (complex a in Figure 5C) or through the catechol moiety (complex b in Figure 5C). Modeling (C3 point group symmetry) and optimization calculations were carried out for low, intermediate and high spin states. The high spin complex was the one with the lowest energy in both models. The calculated UV-Vis spectra are shown in Figure 5B. TD-DFT calculations predicted absorption spectra presenting a band centered at 510 nm of absorbance, for both the a and b complex, originated from a metal to ligand charge transfer transition, in good correspondence with the experimental spectra. Thus, calculations confirmed that both complexes didox-iron are stable but the hydroxaminic group is also present in HU that has no binding affinity, while the catechol moiety is present in DFP that has high iron affinity binding. Thus b seems to be the likely complex.

### 2.5. Didox Alters the Iron Status of HA22T/VGH Cells Similarly to DFO and DFP

**Figure 6:**
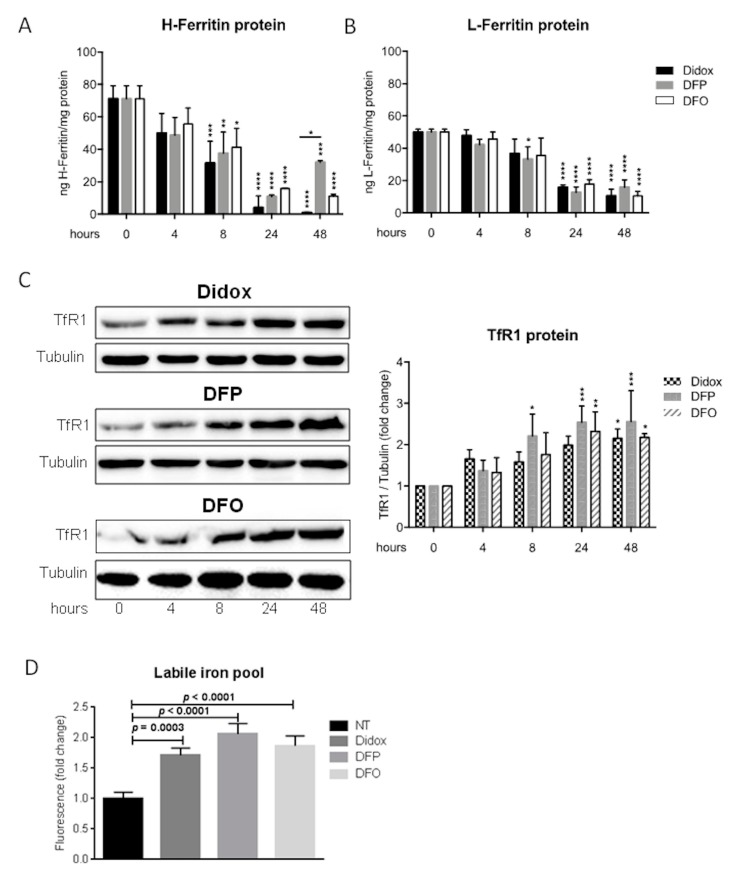
Didox reduced the ferritin level and labile iron pool (LIP) and induced a TfR1 increase in HA22T/VGH cells. (**A**) and (**B**) An ELISA assay for H- and L-ferritin in HA22T/VGH cells treated with didox, DFP and DFO 100 µM at 4, 8, 24 and 48 h; (**C**) TfR1 and tubulin western blotting analysis with didox, DFO and DFP 100 µM at 4, 8, 24 and 48 h; (**D**) calcein-AM assay in cells treated with didox, DFP and DFO 100 µM at 4 h. The graphs are means of three independent experiments (*N* = 3). *p*-values, showed in the graphs, were obtained by an ordinary one-way ANOVA.

It is well known that an iron chelator causes the reduction of both L- and H-ferritin and the labile iron pool (LIP) and the increase of transferrin receptor 1 (TfR1) expression. Thus to verify how didox modifies the iron status of the HCC cells in comparison with DFP and DFO, HA22T/VGH cells were treated with 100 µM Didox, or DFP or DFO for 4, 8, 24 and 48 h and analyzed for L- and H-ferritin content by an ELISA assay (Figure 6A,B) and for TfR1 by western blotting (Figure 6C).

DFO and didox caused a significant and parallel time-dependent reduction of H-ferritin that was maintained up to 48 h, whereas DFP started losing its efficacy at 48 h (Figure 6A). A similar behavior was evident for L-ferritin (Figure 6B). TfR1 increased of about two-fold during the time, in DFO, DFP and didox treated cells, as expected, due to the effect of iron chelation (Figure 6C). LIP was determined by calcein-AM fluorescent assay after 4h of treatment and the values expressed as fluorescence fold change over the untreated cells (Figure 6D). Didox, DFP and DFO caused a similar increase of calcein fluorescence, which indicated a significant and comparable reduction of LIP (Figure 6D). These results suggested that didox iron-chelating activity in the cells is comparable to that of DFO and DFP.

### 2.6. Iron Supplementation Suppresses the Cell Toxicity of Didox in HA22T/VGH

**Figure 7:**
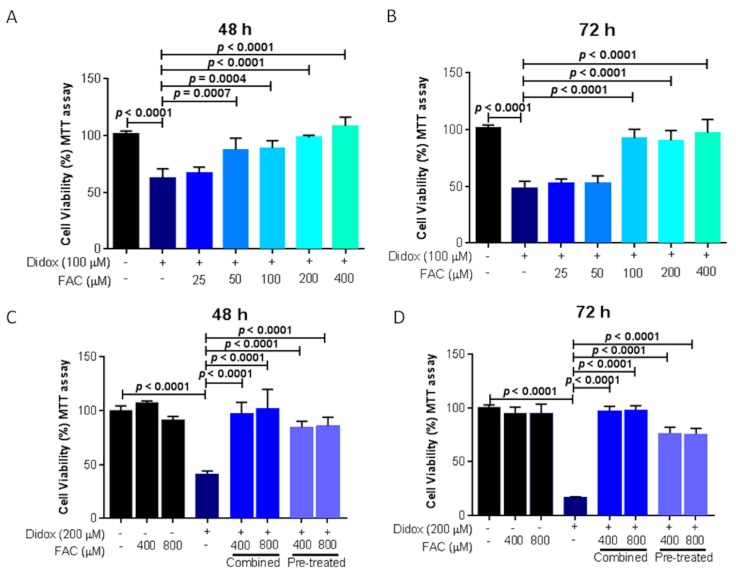
Treatment with equimolar concentration of iron rescued didox-induced cell death. HA22T/VGH cells were untreated or treated with: 100 µM didox alone or in combination with increasing concentration of FAC (25, 50, 100, 200 and 400 µM) for 48 (**A**) and 72 h (**B**). (**C**,**D**) HA22T/VGH cells were untreated or treated with 400–800 µM FAC, 200 µM didox for 48 and 72 h, in combination FAC plus didox (combined) or HA22T/VGH cells were pre-treated with 200 µM didox (for 16 h) and then 400–800 µM FAC was added to the cells (pre-treated) for 48–72 h. Cell viability was verified by an MTT assay and the values expressed as % of viable cells over the untreated cells at the indicated time point. The graphs are means of three independent experiments (*N* = 3) with three internal values for each experiment.

If didox acts as an iron chelator, the addition of iron could decrease or completely abolish the activity of the compound in inhibiting cell viability. To prove that, HA22T/VGH cells were treated with 100 µM didox together with different concentrations of ferric ammonium citrate (FAC, 25, 50, 100, 200 and 400 µM), and the cell viability evaluated after 48 and 72 h with an MTT assay. FAC reduced didox cell toxicity in a dose-dependent manner starting at a concentration of 50 µM at 48 h and of 100 µM at 72 h abolishing the inhibitory activity of didox (Figure 7A,B). With a similar trend, FAC reduced the cell mortality induced also by DFO and DFP starting at concentration of 25 µM both at 48 and 72 h for DFO and DFP (Figure S2A,B). On the opposite, the inhibitory activity of HU was not affected by the iron addition of FAC at all the concentrations and time points tested (Figure S2A,B).

In other experiments the cells were pre-treated with 200 µM didox for 16 h and then 400–800 µM FAC was added and the cells collected after another 48 and 72 h (pre-treatment, in Figure 7C,D), as control didox and FAC were added together (combined, in Figure 7C,D). The iron supplementation suppressed didox activity when added together and also when added after 16 h didox (pre-treatment) with a rescue of about 50–60% at 48 h and 60–70% at 72 h (Figure 7C,D).

**Figure 8:**
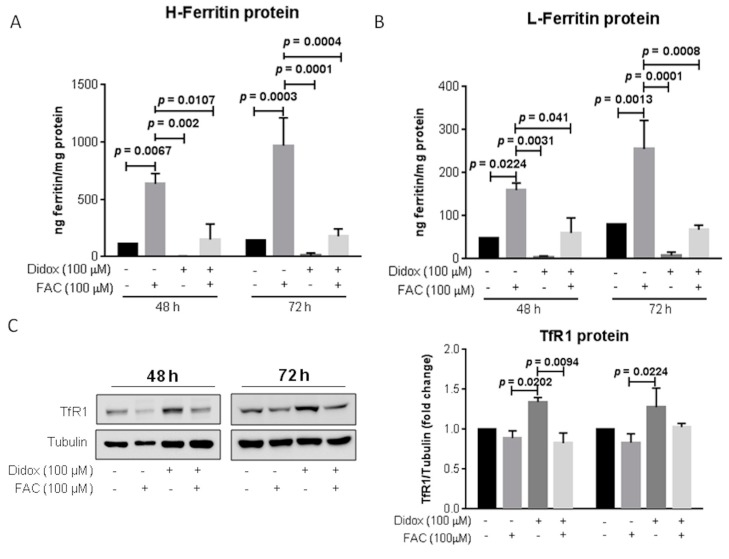
Treatment with equimolar concentration of iron restored iron-related protein content. HA22T/VGH cells were untreated or treated with: 100 µM didox alone or in combination with FAC (100 µM) for 48 and 72 h. Protein extracts were analyzed for H- and L-ferritin content (**A**,**B**) by an ELISA assay and for TfR1 by western blotting (**C**), using tubulin as a loading control. The graphs are the mean of three independent experiments (*N* = 3). *p*-values, showed in the graphs, were obtained by an ordinary one-way ANOVA.

To verify if the iron supplementation inhibits didox activity restoring the proper level of iron related proteins (such as ferritins and TfR1), the HA22T/VGH cells were treated with 100 µM didox or with 100 µM FAC alone or in combination. Didox alone caused a reduction of both H- and L-ferritins (Figure 8A,B) and an increase of TfR1 (Figure 8C) after 48 and 72 h (as previously shown in Figure 6A–C), FAC alone treatment caused ferritin to increase and TfR1 to decrease, as expected. Interestingly, the co-treatment with FAC and didox restored the basal levels of ferritins and TfR1, demonstrating an effect also on iron related proteins connected to the iron binding activity of didox.

## 3. Discussion

Didox has been used for many years as an antitumor agent [14,15,16,17]. It targets and inhibits RRM2, the enzyme involved in the critical conversion of ribonucleotides to deoxyribonucleotides, essential in DNA replication and one of the most expressed enzymes in tumor cells. Didox is a derivative of HU that is known to inhibit RRM2 activity by quenching the tyrosyl free radical at the active site of the enzyme that is essential for the reductase activity [34]. The free radical quenching moiety of HU is partially conserved in didox, and it contains an additional catechol group that is known to have iron-chelating properties. In fact, a major class of bacterial siderophores uses catechol as an iron ligand [35]. The iron-chelating properties of didox were previously studied to define the formation of the iron complex and to show that iron supplementation reduced the cellular toxicity of didox in L1210 leukemia cells [21]. We used the hepatocellular carcinoma (HCC) cell lines, as a cellular model to study didox antitumor activity since HCC is the most common type of liver cancer. HCC are solid tumors, with a large angiogenic capacity and are often resistant to apoptosis and they are classified on the degree of malignancy and the level of differentiation [36]. HCC cells derive from liver, which has a high intracellular iron level, and high expression of iron-related proteins, detectable RRM2 levels and normal proliferation rate. The HuH7 cells are highly differentiated while HA22T/VGH cells are poorly differentiated. We found that these cells are similarly sensitive to didox with an IC_50_ as low as 132 µM in HA22T/VGH and 122 µM in HuH7 after 72 h of incubation. We concluded that the differentiation state did not modify cell sensitivity to the drug, thus we continued the study on the HA22T/VGH cells. We confirmed that didox induced apoptosis also in HA22T/VGH cells with an increase of AnnexinV and mitochondrial ROS production, similar to previous studies reported in multiple myeloma cells [15]. We compared the antitumor activity and iron binding capacity of didox with those of the two clinically used iron chelators, DFP, which is a catechol bidentate structurally similar to didox, and DFO a hexadentate structurally unrelated to didox. When given to the cells, didox caused modifications of the iron status that were very similar in extent to those caused by the two chelators: changes in the level of TfR1 and ferritins and of the intracellular labile iron. Moreover we found that DFO and DFP had a cytotoxic effect on our cells that was comparable to that of didox, and that was relieved by iron supplementation to the cells at concentrations in the same range of that of the chelators. The cytotoxic potency of HU was lower and, more important, not affected by iron supplementation, indicating a different mechanism of action. Iron is essential for many tumor cells, that require it to proliferate, the so-called “iron addition”, thus the iron chelation activity promoted by didox, DFO and DFP inhibits the growth capacity of tumor cells. For example, sub-lethal concentrations of didox affected the capacity of HCC to close the wound (not shown), showing that iron is important also in this process. Our modeling studies indicate that didox binds iron as a bidentate to form a complex didox-iron of 3:1 and the binding probably occurs through the catechol moiety as it occurs in DFP. Didox seems to combine the free-radical scavenging activity of HU that blocks the tyrosil radical of RRM2 with an iron chelating activity of DFP and DFO, which sequesters iron from many key enzymes, among which RRM2 is central. Thus the observation that didox targets RRM2 with two different mechanisms should make it superior to the iron chelators or HU as antitumor agent.

The published data on the use of didox to treat different tumors and our results on HCC in vitro are promising. In vivo studies on HCC, using didox alone or in combination with different chemotherapeutic drugs, could be an interesting point to be defined in the future to finalize the use of this compound as an antitumor drug.

## 4. Materials and Methods

### 4.1. Antibodies and Chemicals

Antibodies used were anti-TfR1 (no. 136800, Thermo Scientific, Waltham, MA, USA) and anti-tubulin (no. T5168, Sigma-Aldrich, Saint Louis, MO, USA). HRP-conjugated secondary antibodies used were anti-mouse (no. sc-516102) and anti-rabbit (no. sc-2357; Santa Cruz Biotechnology, Dallas, TX, USA). The chemicals used in this study were: Didox (3,4 dihydroxybenzohydroxamic acid; no. 10009081, Cayman Chemicals, MI, USA) and hydroxyurea (no. H8627, Sigma-Aldrich, Saint Louis, MO, USA) dissolved in dimethylsulfoxide (DMSO), three different well known iron chelators such as DFO (deferoxamine; no. S0080A, Novartis, Basel, Switzerland), DFP (deferiprone, kind gift of Prof. P. Ponka, University, Montreal, QC, Canada) and BPS (batophenantroline disulfonic acid, no. B1375); FAC (ferric ammonium citrate, no. F5879); FAS (ferrous ammonium sulfate, no. F2262); FeCl_3_ (ferric chloride, no. 157740) and ascorbic acid (no. A4034; Sigma-Aldrich, Saint Louis, MO, USA) all dissolved in water. Calcein (no. 21030 Sigma-Aldrich, Saint Louis, MO, USA), calcein-AM (no. ALX-610-026 Calcein-acetoxymethyl ester, Enzo Life, Lausen, Switzerland) and MTT (thiazolyl blue tetrazolium bromide, no. M5655 Sigma-Aldrich, Saint Louis, MO, USA) were also used.

### 4.2. Cell Culture

The human hepatoma cell lines, HuH7 (from IZSLER, Brescia, Italy), were cultured in Dulbecco modified eagle medium (DMEM; Gibco, Life Technologies, Carlsbad, CA, USA) supplemented with 10% endotoxin-free fetal bovine serum (Gibco, Life Technologies, Carlsbad, CA, USA), 0.04 mg/mL gentamicin (Gibco, Life Technologies, Carlsbad, CA, USA), 2 mM L-glutamine (Gibco, Life Technologies, Carlsbad, CA, USA) and 1 mM sodium pyruvate (Carlo Erba, Milan, Italy). The HA22T/VGH cell lines, a kind gift of Dr. A. Salvi and Prof. G. De Petro (University of Brescia, Brescia, Italy), were maintained in RPMI-1640 (Gibco, Life Technologies, Carlsbad, CA, USA) supplemented with 10% endotoxin-free fetal bovine serum, Fungizone (Gibco, Life Technologies, Carlsbad, CA, USA), 0.04 mg/mL gentamicin (Gibco, Life Technologies, Carlsbad, CA, USA), 2 mM L-glutamine (Life Technologies, Carlsbad, CA, USA) and 1 mM sodium pyruvate (Carlo Erba, Milan, Italy). The cell lines were maintained at 37 °C in a 5% CO_2_ incubator.

### 4.3. Cell Treatments and Cell Viability Analysis

The cells were seeded in a 96-well plate (at a density of 2 × 10^3^ cells for HA22T/VGH; 1.5 × 10^3^ cells for HuH7) and exposed to various concentrations of didox and only HA22T/VGH also to hydroxyurea, DFO or DFP (0, 1, 10, 25, 50, 100, 200 and 500 µM) for 24, 48 and 72 h. In other experiments, HA22T/VGH were seeded in 96-well plates and treated with a single dose of didox, HU, DFO, DFP alone or in combination with increasing doses of FAC (25, 50, 100, 200 and 400 µM) for 48–72 h. In other type of treatment, HA22T/VGH cells were or pre-treated for 16 h with a single dose of didox (200 µM) and then treated in combination with FAC (400–800 µM) or directly in combination didox-FAC for 48–72 h.

Cell viability was evaluated with an MTT assay (Sigma-Aldrich, Saint Louis, MO). After the indicated time points and treatments, the supernatant was removed and 100 µL of the MTT solution (0.5 mg/mL) diluted in the cell medium was added to the wells. After 3.5 h of incubation at 37 °C and 5% CO_2_, the MTT medium was removed and 75 µL of DMSO was added to each well. Plates were shaken for 15 min at 37 °C until complete dissolution and absorbance was measured at 540 nm emission wavelengths using a Multiskan^©^EX plate reader (Thermo Scientific, Waltham, MA, USA). Average percentage of cell viability at each concentration was calculated using Microsoft Excel 2016 software.

### 4.4. Protein Extraction

Cells extracts were prepared using a lysis buffer (200 mM Tris-HCl pH 8, 100 mM NaCl, 1 mM EDTA, 0.5% NP-40, 10% glycerol, 1 mM sodium fluoride and 1 mM sodium orthovanadate; Complete Protease Inhibitor Cocktail; Sigma-Aldrich). The protein concentration was quantified using Micro BCA^TM^ Protein Assay Kit (Sigma-Aldrich, Saint Louis, MO, USA) and used for different analysis by western blotting and ELISA assays.

### 4.5. Western Blot Analysis

Western blot was used to analyze protein expression. In brief, after extraction, equal amounts of protein homogenates were boiled at 99 °C for 5 min before separation by SDS–polyacrylamide gel electrophoresis and transferred to a polyvinylidene fluoride (PVDF) membrane (GE healthcare, Little Chalfont, UK). Membranes were blocked for 30 min at 37 °C with Tris-buffered saline with 1% Tween-20 (TBS-T) with 2% milk and incubated overnight at 4 °C or 2 h at 37 °C with the primary antibodies (reported in the material and methods paragraph). Following the TBS-T wash, membranes were incubated with HRP-conjugated secondary antibodies for 1 h and 30 min at RT. Membranes were washed again in TBS-T prior to signal visualization using enhanced chemiluminescence (PDS kit, Protein Detection System, GeneSpin, Milan, Italy). The signal was visualized with a Lycor Odyssey instrument and densitometric analysis was performed using ImageJ software (NIH, Bethesda, MD, USA) and normalized against tubulin, as a loading control.

### 4.6. ELISA Assay

The plates (96 wells) were coated with 0.1 mL of primary antibody against L-ferritin (LFO3) or H-ferritin (RH02; 10 μg/mL diluted in 50 mM carbonate buffer pH 9.6) for 18 h at 4 °C. After three washes with PBS-T (phosphate buffer saline with 0.05% Tween20), the wells were over-coated by adding 0.1 mL of 3% bovine serum albumin (BSA) diluted in PBS for 30 min at 37 °C. After washing with PBS-T, 20 μg of protein extract for L-ferritin and 5 μg of protein extract for the H-ferritin analysis were aliquot in duplicate, diluted in 1% BSA-PBST and incubated at 37 °C for 2 h. A standard curve using recombinant human L- or H-ferritin was added into the plate, as a calibrator. After three washings in PBST, 0.1 mL of anti-L- or H-ferritin antibody HRP labeled (diluted 1:500 in 1% BSA-PBS, respectively) were added and plate incubated for 1 h at 37 °C. After three washings in PBS-T, HRP activity was detected using 1 mg/mL tetramethylbenzene (TMB) dissolved in dimethyl sulfoxide (DMSO) and diluted 1:10 in phosphate-citrate buffer pH 5 with added fresh hydrogen peroxide to a final concentration of 0.006% and the absorbance read at 620 nm by Multiskan^©^EX plate reader. The reaction was stopped by adding 1 N sulphuric acid and the absorbance was measured at 405 nm. The concentration of ferritins was extrapolated from the calibrator curve and expressed as ng of ferritin/mg of protein extract.

### 4.7. Labile Iron Pool Assay and Calcein-AM Assay

The cellular LIP was measured as described elsewhere, with minor modification [37]. Briefly, HA22T/VGH cells (3 × 10^4^) were seeded on 96-well plates and treated with 100 µM DFO, DFP or didox for 4 h. The cells were incubated with 0.25 µM calcein-AM in MEM with 1 mg/mL BSA for 30 min at 37 °C. After washing with 1X phosphate-buffered saline (PBS), 100 μL of 1X Hank’s Balanced Salt Solution (HBSS) was added to the cells and the fluorescence was monitored at an excitation of 488 nm and an emission of 517 nm using an EnSight Multimode plate reader (Perkin Elmer). Cells were then fixed in 4% PFA, stained with crystal violet solution (0.1% crystal violet, 20% methanol) for 15 min. After washings, 100 μL of 10% acetic acid was added and absorbance was detected at 540 nm using a Multiskan^©^EX plate reader (Thermo Scientific, Waltham, MA, USA). The data were expressed as fold change over the not treated cells (ratio of fluorescence of calcein-AM/absorbance at 540 of crystal violet). The quenching of calcein-AM is inversely proportional to the concentration of intracellular iron.

### 4.8. MitoSOX™ Red Mitochondrial Superoxide Indicator Assay

HA22T/VGH cells were seeded in six-well plates and 24 h after seeding, treated with 200 µM didox, respectively. After 24, 48 and 72 h, cells were collected and labeled with 5 µM MitoSOX™ (Molecular Probes) diluted in medium and incubated in the dark for 20 min at 37 °C. Cells were then washed and suspended in medium and fluorescence detected by a citofluorimeter instrument (MACSQuant Analyzer, Miltenyi Biotec, Germany).

### 4.9. AnnexinV/Propidium Iodide Assay

HA22T/VGH cells were seeded in six-well plates (250 × 10^5^ cells/well). Twenty-four hours after the seeding, cells were untreated or treated with 200 µM didox and the apoptotic cell death analyzed after 24, 48 and 72 h using the commercial kit AnnexinV-FITC Apoptosis Detection (Immunostep) and following the manufacturer’s instructions. Briefly, cells were harvested, washed with 1X PBS and resuspended in 1X Annexin-binding buffer followed by the addition of 5 μL of AnnexinV-FITC and 5 μL of propidium iodide (PI). The cells were then incubated at RT for 15 min in darkness. After incubation, 400 μL of 1X Annexin-binding buffer was added and cells analyzed by flow cytometry within one hour, using the MACSQuant Analyzer (Miltenyi Biotec). Analysis of apoptosis was performed by counting cells stained simultaneously with AnnexinV–FITC and PI, to discriminate intact cells (AnnexinV-FITC and PI negative) from cells in the early apoptotic state (AnnexinV-FITC positive and PI negative) and late apoptotic state (AnnexinV-FITC and PI positive). The percentage of cells positive to each dye was represented in the plot, whereas the histogram showed the cells positive to AnnexinV-FITC (the sum of the percentage of cells in early and late apoptosis).

### 4.10. Dequencing of Calcein Fluorescence In Vitro

Calcein fluorescence (excitation 488 nm; emission 517 nm) was detected by an EnSight Multimode plate reader (Perkin Elmer), following the protocol from Breuer W. et al. (1995) [32]. Briefly, 1 μM of calcein (Sigma-Aldrich, Saint Louis, MO, USA) was incubated with an equimolar concentration of iron (II), as 1 μM FAS (ferrous ammonium sulfate) and allowed to equilibrate for 8 min before analyzing the quenching of fluorescence. Iron (III) chelators such as DFO and DFP, or iron (II) chelator such as BPS, or didox or HU were subsequentially added at the final concentration of 100 μM. The dequenching of calcein fluorescence was analyzed after 1, 2, 3, 4, 5, 15, 25, 35, 45, 55 and 65 min. 

### 4.11. UV-Vis Spectroscopy 

The absorption spectra of didox combined with iron (III) were measured with a Jasco 815SE instrument. Conditions were 2-nm resolution, 200 nm/min scan speed and 1 cm quartz cuvette. A solution of 300 µM of ferric chloride was prepared in the Tris-HCl buffer, 25 mM, pH 7.2. Increasing amounts of didox (from 0 to a final concentration of 900 µM) were added to the solution. After each addition of didox, the solution was left for 30 min at 25 °C, then UV-vis spectra were recorded.

### 4.12. Computational Method

Geometry optimizations and UV-vis spectra calculations were performed with the use of the Gaussian16 program (Gaussian 16, Revision B.01, M. J. Frisch 2016), with CAM-B3LYP functional and 6 − 31 + G** basis set and MDF10 pseudopotential in the IEF-PCM approximation for the solvent. The 0.2 eV broad Gaussian band-shape was used.

### 4.13. Statistical Analysis

Data are presented as mean ± standard error of mean (SD). Statistical significance was assessed by a two-way ANOVA, unless otherwise indicated in the figure legends, and performed by GraphPad Prism 5 (GraphPad Software, Inc., La Jolla, CA). *p*-values < 0.05 were considered significant.

## Supplementary Materials

The following are available online at https://www.mdpi.com/1424-8247/12/3/129/s1, Figure S1: Didox reduced cell viability in the HCC cell line in time and a dose-dependent manner. Table S1: Calculation of IC_50_, Figure S2: Treatment with an equimolar concentration of iron rescued DFO- and DFP- but not HU-induced cell death.
