# Supplementary material for: The Antitumor Didox Acts as an Iron Chelator in Hepatocellular Carcinoma Cells
Source: Pharmaceuticals (Basel). 2019 Sep 2;12(3):129. doi: 10.3390/ph12030129 (PMC6789654; doi:10.3390/ph12030129)
Supplement: Supplementary file 1 [file pharmaceuticals-12-00129-s001.pdf]

Supplementary Materials

Figure S1

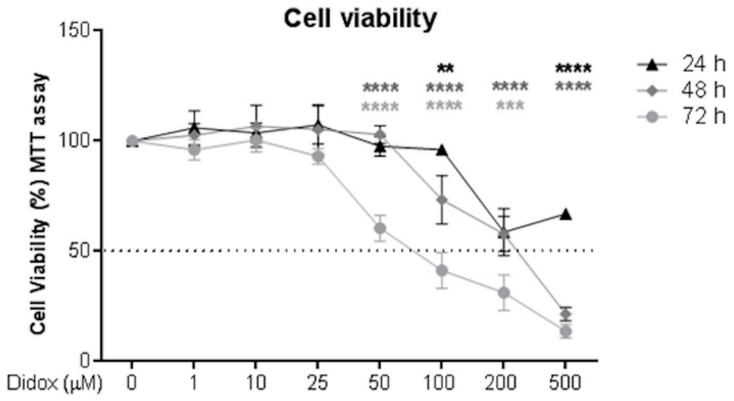

Figure S1. Didox reduced cell viability in the HCC cell line in time and a dose-dependent manner. HuH7 were treated with 0, 1, 10, 25, 50, 100, 200 and 500 μM didox for 24 (triangle, black line), 48 (diamond, grey line) and 72 (circle, light grey line) hours. An MTT assay was performed to verify cell viability after treatment. The values are expressed as % of viable cells over the not treated cells at the indicated time point. The black dot line is drawn in correspondence to the half maximal inhibitory dose (IC_{50}). The graph is the mean of three independent experiments ($N = 3$) with three internal values for each experiment. The black stars correspond the comparison between 24 and 48 h; the grey stars between 24 and 72 h and the light grey stars between 48 and 72 h. *: $p < 0.05$; **: $p < 0.01$; ***: $p < 0.001$; ****: $p < 0.0001$.

Table S1

	**IC_{50} (μM) 48 h**	**IC_{50} (μM) 72 h**
**HA22T/VGH**	283.36 ± 18.82	132.98 ± 7.97
**HuH7**	329.31 ± 31.55	122.92 ± 13.21

Table 1. Calculation of IC_{50}. For HA22T/VGH and HuH7 cell lines the IC_{50} (the dose of the compound, which was reduced by 50% of the cell viability) was calculated at 48 and 72 h of treatment with didox. The data, derived from the graphs in Figure 1; Figure 1S, are the mean of three independent experiments.

Figure S2

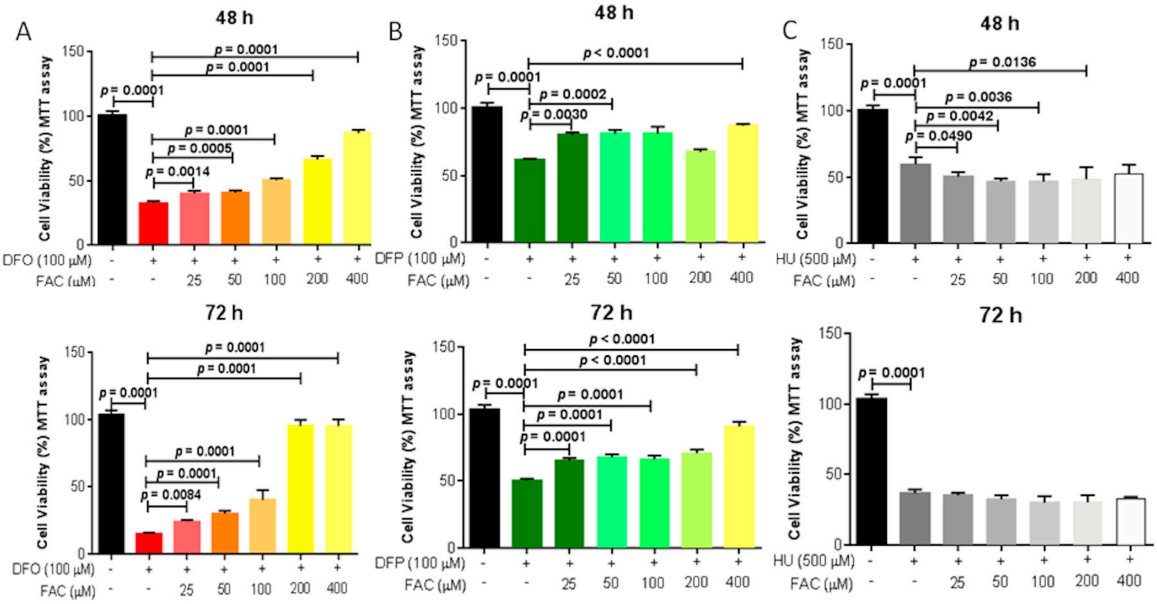

Figure S2. Treatment with an equimolar concentration of iron rescued DFO- and DFP- but not HU-induced cell death. HA22T/VGH cells were untreated or treated with: 100 µM DFO (**A**), DFP (**B**) or 500 µM HU (**C**) alone or in combination with increasing concentration of FAC (25, 50, 100, 200 and 400 µM) for 48 (up) and 72 h (down). Cell viability was verified by an MTT assay and the values expressed as % of viable cells over the untreated cells at the indicated time point. The graphs are the means of three independent experiments (*N* = 3) with three internal values for each experiment.
